# Coparenting Quality During COVID-19: Exploring Gender Differences Using a Mixed Methods Approach

**DOI:** 10.1177/0192513X241236555

**Published:** 2024-02-29

**Authors:** Sabrina Douglas, Katherine Morrison, Alison Miller, Jess Haines

**Affiliations:** 1 Department of Family Relations and Applied Nutrition, 3653 University of Guelph, Guelph, ON, Canada; 2 Centre for Metabolism, Obesity & Diabetes Research, Department of Pediatrics, 3710 McMaster University, Hamilton, ON, Canada; 3 Department of Health Behaviour and Health Education, School of Public Health, 1259 University of Michigan, Ann Arbor, MI, USA

**Keywords:** COVID-19, coparenting, families, gender, thematic analysis

## Abstract

The aim of this study was to examine potential differences in coparenting quality during the COVID-19 pandemic among mothers and fathers using an embedded mixed methods approach. The objectives were to compare mothers’ and fathers’ scores on the Coparenting Relationship Scale among 150 mother–father dyads, and to examine mothers’ and fathers’ perceptions of how COVID-19 influenced their coparenting quality using thematic analysis of 159 mothers’ and 75 fathers’ responses to an open-ended coparenting survey question. While total coparenting quality scores did not differ among mothers and fathers, fathers had significantly higher scores on the division of labour and endorsement subscales, and mothers had significantly higher scores on the undermining subscale. The qualitative thematic analysis identified five key themes: gendered changes to division of labour, increased hostility, increased teamwork, less alone time, and increased stress. Efforts to mitigate adverse pandemic outcomes on families should address coparenting quality.

## Introduction

The COVID-19 pandemic resulted in large-scale disruptions to home life, businesses, child care, and schools in Canada. Within the first few months of the pandemic, Canada experienced a 15% decline in employment (Lemieux et al., 2020) and nearly 40% of Canadians were working from home (Canada, 2022). Women tended to work from home more than men (Canada, 2022) and women’s employment was disproportionately affected compared to men, especially among women with children (Fuller & Qian, 2021). Disruptions to child care and schools led to high levels of stress and adverse consequences to family functioning and well-being, particularly among families with preschool aged children (Carroll et al., 2020; Gadermann et al., 2021; Prime et al., 2020).

Coparenting quality, defined as the way the parents work together and support each other in their roles as parents or caregivers (Feinberg, 2003), is an important aspect of family systems that was also adversely impacted by the pandemic (Feinberg et al., 2022) as parents tried to jointly navigate unprecedented circumstances while caring for their children. The Coparenting Relationship Scale (CRS), developed by Feinberg et al. (2012), is a widely used tool for assessing coparenting quality, and includes items that measure parents’ perception of seven aspects of coparenting quality: (1) Coparenting agreement refers to the level of agreement between parents on matters related to their child; (2) Coparenting support refers to parents’ perception of the level of support they receive from their partner; (3) Division of labour refers to a parents’ perception of their partner’s contribution to parenting responsibilities; (4) Closeness refers to parents’ perception of how parenting has brought them and their partner closer as a couple; (5) Endorsement of partner’s parenting refers to a parents’ perception of their partner’s parenting; (6) Conflict refers to how much parents engage in conflict in the presence of their child; and (7) undermining refers to parents’ perception of how much they are undermined by their partner. A higher coparenting quality score indicates a more favourable perception of coparenting quality and is characterized by greater agreement, support, division of labour, closeness, and endorsement of partner’s parenting, and less conflict and undermining (Feinberg et al., 2012).

A study by Feinberg et al. (2022) compared American parents’ coparenting quality before the pandemic and during the pandemic and reported decreases in both mothers’ and fathers’ coparenting quality. While the Feinberg study did not examine differences in coparenting quality between mothers and fathers, there is some evidence that the COVID-19 pandemic may have differentially impacted coparenting quality among mothers versus fathers. Division of labour may have been particularly susceptible to gendered effects caused by the pandemic due to changes in work hours, working from home, and accessibility of child care. There is some evidence that fathers were more involved in household tasks and child care at the beginning of the pandemic (Shafer et al., 2020); however, a study of mothers and fathers in similar career roles in Canada found that mothers reported having less time to focus on work and spent more time caring for children compared to fathers (Gordon & Presseau, 2023). Research also suggests that the protective effects of coparenting support may be gendered. Two studies found that fathers’ reports of coparenting support and cooperation were found to mitigate the effects of pandemic-related stressors on their mental health; however, this association was not found among mothers (Bastiaansen et al., 2021; He et al., 2021). Understanding whether coparenting quality scores during COVID-19 differ between mothers and fathers may help explain some of these differential impacts of pandemic-related stressors on mothers versus fathers and can help inform intervention and policy efforts to mitigate adverse pandemic outcomes among families.

While quantitative methods allow for an examination of how coparenting quality scores may differ between mothers and fathers, qualitative methods can provide a more in-depth and nuanced understanding of parents’ perceptions of specific coparenting domains affected by the COVID-19 pandemic (Choy, 2014; Kotila & Schoppe-Sullivan, 2015). A recent qualitative study in the UK examined mothers’ perception of COVID-19-related coparenting during COVID-19 and found that mothers perceived inconsistent support and lack of engagement from their partner, as well as positives such as better communication, more coparenting involvement and increased closeness (Cox et al., 2023). Few studies have explored fathers’ perception of coparenting quality during the COVID-19 pandemic. Thus, the aim of this study was to examine potential differences in coparenting quality among mothers and fathers using an embedded mixed methods approach (Creswell & Plano Clark, 2011). The first objective of this study was to compare mothers’ and fathers’ scores on seven domains of coparenting and overall coparenting quality assessed using the CRS during the COVID-19 pandemic. Based on previous findings related to coparenting (Douglas et al., 2021), we hypothesized that compared to fathers, mothers would report lower total coparenting quality scores and lower coparenting subscale scores, in particular on the division of labour subscale. The second objective was to examine mothers’ and fathers’ perceptions of how coparenting changed during the COVID-19 pandemic by conducting a thematic analysis of parents’ responses to an open-ended coparenting survey question. We hypothesized that compared to fathers, mothers would report greater challenges in their coparenting relationship due to COVID-19.

## Methods

### Study Design and Participants

We conducted an embedded mixed methods study using data from parents’ responses on the baseline survey of the Family Stress Study, a prospective cohort study of families with children aged two to six years designed to examine the impact of chronic stress on children’s outcomes. Families were eligible to participate if they had at least one child in the target age range and did not plan to move from the Guelph/Hamilton area within three years. Families were recruited primarily through online social media, and through posters in family centered areas of the community (e.g., libraries and recreation centers). Data were collected from July 2020 to December 2021. The study was approved by the University of Guelph Research Ethics Board (REB1911047) and the Hamilton Integrated Research Ethics Board (10763).

### Procedures

#### Objective 1

To compare mothers’ and fathers’ scores on seven domains of coparenting and overall coparenting quality assessed using the CRS during the COVID-19 pandemic.

##### Measure

Mothers and fathers completed the brief version of the Coparenting Relationship Scale (CRS; Feinberg et al., 2012). This measure includes 14 items that assess seven aspects of coparenting quality, including coparenting agreement, coparenting closeness, coparenting support, endorsement of partner’s parenting, division of labor, exposure to conflict, and coparenting undermining. Items were rated on a 6-point Likert scale and a total score was created by averaging the responses to all the items on the brief version of the survey. Higher subscale scores indicate greater endorsement of the subscale. Seven items were reverse scored to ensure that higher total coparenting quality scores indicated better coparenting quality. The Spearman–Brown coefficient has been identified as an appropriate test to measure internal consistency among 2-item scales (Eisinga et al., 2013); therefore, we calculated the Spearman–Brown coefficient for each two-item subscale (values ranged between 0.57–0.89), and Cronbach’s Alpha for the total coparenting score (Cronbach’s alpha = 0.87).

##### Statistical Analysis

Statistical analyses were performed using IBM SPSS Statistics (version 28). Wilcoxon signed-rank test of differences were used to test for differences between mothers and fathers on the CRS subscale scores and overall score. Wilcoxon signed-rank tests were used because parents’ responses on the CRS were not normally distributed and because the two samples were not considered independent since mothers and fathers are in a relationship and cohabitating. *p*-values less than 0.05 were considered statistically significant.

#### Objective 2

To examine mothers’ and fathers’ perceptions of how coparenting changed during the COVID-19 pandemic by conducting a thematic analysis of parents’ responses to an open-ended coparenting survey question.

##### Measure

Participants responded to an optional open-ended survey question, administered after the CRS, which asked participants “How has the way you and your partner work together as parents changed since COVID-19?”.

##### Analysis

NVivo (release 1.7.1) was used to organize and analyze the qualitative data from the open-ended coparenting survey question. The first author conducted codebook thematic analysis according to Braun and Clarke (2022). The first stage of coding was *familiarization*, where the researcher read all parents’ responses to gain an understanding of interesting trends and to take notes to help guide the rest of the analysis (Braun et al., 2019). The second stage was *code generation* which involved labeling responses using an inductive coding approach, that is, codes were developed during the analysis instead of prior to analysis (Braun et al., 2019). Each response was assigned a code, which helped organize the data into different topic areas. The final stage of analysis was *theme construction* which involved using the codes generated in the previous stage to help identify major themes across the data.

## Results

### Sample

Participant characteristics of the quantitative (objective 1) and qualitative (objective 2) samples are presented in Table 1. A total of 359 families participated in the Family Stress Study (FSS), from which these samples were drawn. Sociodemographic characteristics of the quantitative and qualitative samples were similar. Parents’ mean age was 36–37 years. Most parents graduated from college or university, identified as white, and indicated they had two children in their household.

**Table 1.** Summary of Parent and Family Sociodemographic Characteristics of the Quantitative Sample (*n* = 150 Mothers, 150 Fathers, 150 Families) With Responses to the Coparenting Relationship Scale and Qualitative Sample (*n* = 159 Mothers, 75 Fathers, 199 Families) With Responses to the Open-Ended Coparenting Survey Question.

	Quantitative sample *n* (%)	Qualitative sample *n* (%)
Relation to child		
Mother	150 (50.0)	159 (67.9)
Father	150 (50.0)	75 (32.0)
Number of children per family^a^		
1	25 (16.7)	45 (22.6)
2	93 (62.0)	117 (88.9)
3 or more	32 (21.3)	37 (18.6)
Parent age in years, mean (SD)	36.69 (4.61)	35.88 (5.06)
Parent education^b^		
Less than a college/university education	42 (14.0)	30 (12.8)
Graduated college/university	257 (86.0)	204 (87.2)
Parent race/Ethnicity		
White	247 (82.3)	190 (81.2)
South Asian	13 (4.3)	8 (3.4)
Chinese	7 (2.3)	7 (3.0)
Other (Southeast Asian, West Indian, Black, Aboriginal/Indigenous, Latin American, Korean, or Japanese)	33 (11.0)	29 (12.4)

^**a**^Number of children missing for one participant in the qualitative sample.

^**b**^Education missing for one participant in the quantitative sample.

#### Objective 1

Families with two participating parents who each responded to the CRS (*n* = 150 families, *n* = 150 mothers and 150 fathers) were included in objective 1.

#### Objective 2

FSS families in which at least one parent (mother or father) responded to the open-ended coparenting question were included in objective 2. A total of 182 FSS mothers and 93 FSS fathers responded to this question. Responses from 23 mothers and 18 fathers were removed for not having sufficient data for analysis. Therefore, the analytic sample included 159 responses from mothers and 75 responses from fathers (*n* = 199 families). Responses included 121 families with responses from one mother, 43 families with responses from 1 father, 30 families with responses from a mother and father, 4 families with responses from 2 mothers, and 1 family with responses from 2 fathers.

### Objective 1: Comparisons Between Mothers and Fathers on the Coparenting Relationship Scale

Mean scores for mothers and fathers on the CRS subscales and the total coparenting score are presented in Table 2. While total coparenting quality scores did not differ between mothers and fathers (Z = 0.67, *p* = .50), compared to mothers, fathers had significantly higher scores on division of labour (Z = 4.61, *p* < .05) and endorsement of partner’s parenting (Z = 3.75, *p* < .05), indicating that, compared to mothers, fathers perceived a more equal division of parenting duties between themselves and their partner, and they endorsed their partners’ parenting more positively. Mothers had significantly higher scores than fathers on undermining (Z = −3.56, *p* < .05) suggesting that mothers perceived more undermining from their partners as compared to fathers. Differences between mothers and fathers on agreement, closeness, support, conflict subscales were not statistically significant.

**Table 2.** Mothers’ (*n* = 150) and Fathers’ (*n* = 150) Mean Scores on the Coparenting Relationship Scale and Wilcoxon Signed Tank Test of Differences.

Coparenting variable	Mothers			Fathers			Wilcoxon signed rank test Z-Statistic
	Mean (SD)	Min	Max	Mean (SD)	Min	Max	
Agreement	4.54 (1.26)	0.5	6.0	4.57 (1.27)	0.0	6.0	0.29
Closeness	4.75 (1.46)	0.0	6.0	4.69 (1.31)	0.0	6.0	−0.73
Division of labour	4.21 (1.57)	0.0	6.0	5.02 (1.21)	0.5	6.0	4.61^a^
Support	4.84 (1.44)	0.0	6.0	4.53 (1.41)	0.0	6.0	−1.82
Endorsement of partner	5.37 (0.82)	1.0	6.0	5.66 (0.63)	2.0	6.0	3.75^a^
Undermining	5.37 (0.94)	1.5	6.0	4.86 (1.38)	0.0	6.0	−3.56^a^
Conflict	5.25 (0.87)	0.0	6.0	5.28 (0.83)	0.0	6.0	0.46
Total Score	4.90 (0.86)	1.8	6.0	4.95 (0.77)	2.1	6.0	0.67

Higher subscale scores indicate greater endorsement of the subscale. Higher total coparenting quality scores indicated better coparenting quality. Sample sizes differ slightly due to missing data. Undermining and conflict items reverse scored for calculating the total score.

^**a**^Statistically significant at *p* < .05.

### Objective 2: Thematic Analysis

We identified five themes from mothers’ and fathers’ responses to the open-ended coparenting survey question. The five themes identified in this study are described below and are included in Table 3 along with supplementary quotes illustrating each theme. Due to the complex and dynamic nature of the first theme, we divided this theme into three subthemes, whereas themes two through five did not require specific subthemes.

**Table 3.** Supplementary Quotes From Parents in Response to the Open-Ended Coparenting Question “How has the Way You and Your Partner Work Together as Parents Changed Since COVID-19?” Organized by Theme and Subtheme.

Theme	Subtheme	Description of theme	Quotes
1. Changes in division of labour due to changes in work schedules, job loss, and working from home	Gendered differences in family and work responsibilities	Families report gendered differences in child care and work responsibilities, with mothers often being reported as the main caregiver in families. Mothers also reported a lack of support from partner regarding child care and household labour	“My wife is the main caregiver at home. Sometimes she complains a lot and she is not happy about my parenting skills.” “When he gets home he is tired and does his own thing.” “Been very stressful because I had to stay home and home school our child. He did not have the option to work from home. As the mother/wife I was doing majority of all the house chores and schooling. Unfortunately after the 2nd wave of Covid and home schooling I became so overwhelmed. I had to seek medical help. My children always came first.” “She’s on maternity leave now, but when she was still working we had to arrange our schedules so she could work from home after COVID shut down schools.”
	More shared home responsibilities between parents	Mothers and fathers reported that more time at home has allowed for more shared responsibilities between parents. Parents also mentioned that the parent who used to work outside the home is now able to see firsthand the experiences of the stay-at-home parent	“We were both working from home, which allowed for more responsibility sharing.” “Since he has been home from work, he has been able to be an even more involved parent and see the work that goes into being a stay at home parent.” “I’ve had much more time with my kids since working from home and now I feel as though this has allowed me to offer more help in the day-to-day parenting where I’d normally have been at the office.” “At the beginning I was home with both our children while my partner worked outside of the home. It was very challenging. Now he is off work and able to help out more, I think it has been a good role reversal for us and helps us both appreciate the others experiences over the last 5 years (2 mat leaves, going back to work, Covid lock down)” “With him being home more, it has been very validating for him to see what is involved in the household labour”
	Challenges with routine and renegotiating division of labour	Mothers reported that having their partner home more often disrupted their established home routines, causing challenges to their parenting and renegotiations around division of labour	“My husband has retired and is transitioning to a new role in the home. He is home all the time. It has been helpful during this season for us, but in some ways prevented a 'routine' from being started given all the other transitions…(home school, etc).” “It’s become a lot more difficult because my partner was home a lot due to Covid shut downs so my partner would have more objections to my parenting and vice versa.” “Partner has been interfering more due to joblessness” “My husband used to travel and has been home continuously since March 2020 so we had to figure out routine again.” “Its stressful knowing he’s home but not able to help, and on top of that i need to keep everyone quite while he works and even more quiet. during my daughters online classes. Which is why [child] gets as much screen time as he does to keep him quiet and entertained while i keep my 2 year old entertained.” “More stress...being at home causing more issues with dad, who is used to a lot of alone time.”
2. More hostility between parents		Disagreements between parents about public health recommendations, parents are more annoyed with each other and engaging in more arguments. Hostility was often reported to be due to being “couped up” and not having enough personal space, or due to inequitable changes in division of labour	“I am more aware of COVID and cautious about not getting it and my partner does not have those same feelings… So its hard parenting with someone when the health of your child is at risk. We have also been fighting a LOT more!” “COVID has put a bit more of a strain on our coparenting relationship as we do not follow the same beliefs. I am more cautious regarding COVID and have limited who we see and play with while her father has not.” “We get on each others' nerves more because one or the other of us has to parent 24/7.” “There have been a lot of additional stressors in terms of trying to balance everything else in our lives with having two kids at home full time which does lead to arguments and negative feelings.” “We are always in each others space and that can be frustrating for both of us.” “My husband is essential and I don’t think he understands how difficult it is to parent during this pandemic without his help each day.” “Husband was laid off and I was working from home- much more face time and challenges being in the house so often.” “More stressed *n* cooped up in one place” “We are in a very small space together near constantly and don’t have much room to be alone” My partner is home with us more now so it further illustrates that he does not play an active role at all in parenting or household chores “I still get frustrated because I feel like I hold a lot more of the 'household manager' roles than he does. I also get frustrate because so. many. people tell us how great he is as a dad, when, really, he’s just an equal coparent - shouldn’t that be the standard for dads??”
3. Increased teamwork among parents		Parents were forced to rely on each other more, have better communication, engage in more coordination, and manage child care and work by “tag-teaming”	“We’ve had to rely on each other more than ever and really face some underlying issues that were in the background.” “We used to do more things as a family with all of us. Now we often parent in shifts to give each other a break.” “We do much more “divide and conquer” parenting then being together as a family for anything.” “We have essentially each become single parents as we are working alternative days, 6 days of the week. We have really come to realize how much we rely on each other when we are together to give breaks and take the load off when we can.” “We are more likely to split up time: “Can you take them for 2 hours while I relax, and then we’ll switch?”, etc.” “Clear communication and listening has become even more important especially since spending so much time together since March. We have also become better at sharing what we need eg. 30 mins of alone time, no kids, extra hour to work, etc.” “We definitely spend more time together now, so we’ve had to have increased parent check ins after the kids go to bed, just to insure we’re on the same page.” “Yes - we were very strong and communicative in the start when we were all locked down, but as things progressed and I worked more and [partner] worked more, it started to get harder.” “Without having access to activities and programs has forced us to become better at planning together.”
4. More whole family time, but less time for couple’s relationship		Parents reported that they had more time to spend together as a family, however they had less quality time together alone as a couple	“During the summer, we tried to do a nightly walk once finished dinner. This isn’t something we used to do before, but it has been good for our family to make sure we have tech free time together.” “We have more family time now since cOVID-19. I Guess its a good thing for family relationship?” “We have really appreciated the extra family time as a silver lining during these tough times.” “It has been a challenge balancing household responsibilities - we are both tired at the end of our days and on weekends want to relax together.” “At the beginning of the pandemic I needed to change my work hours in order for both my wife and I to continue working while also caring for [child]. This resulted in us having very little time together outside of weekends.” “We have both been home more together and have put more focus on doing activities as a family.” “No alone time like we had before due to online schooling of our older daughter.” “But at the same time we had virtually no alone time together as one of us was always working which became very stressful and depressing.” “The lack of access to outside help/child care has meant we have less time for things outside of our child (including ourselves and each other).”
5. More stress among parents		Parents reported several types of stress including financial stress due to job loss or one parent having to quit their job to care for children, child care stress due to the unpredictability of daycare closures and illness, and health stress among parents of high-risk children	“Being a stay-at-home mom now, I rely on my partner for most of the expenses to be paid. It has put a lot of financial pressure on him. I used to make equivalent to his income, so we are essentially making 50% of the income we were prior to the coronavirus outbreak.” “We had no access to parenting supports during lockdown and partner had lost his job while I was on parental leave, causing financial stress and alternating how we worked together as parents because we were both so stressed.” “We’re more stressed about his exposure.” “Been very stressful because i had to stay home and home school our child.” “More financial stress”

### Theme 1: Gendered Changes in Division of Labour Due to Changes in Work Schedules, Job Loss, and Working From Home

The most robust theme we found among parents’ responses was related to division of labour often due to changes in work schedules (e.g., working more hours or reduced hours), one or both parents being laid off, or one or both parents working from home. Within this theme we identified three subthemes.

The first subtheme is *gendered differences in family and work responsibilities.* Both mothers and fathers reported that the burden of child care was often mothers’ responsibility, despite both parents working full time. One mother wrote: “Increased burden on me (mom) during school and daycare closure. As the lower earner, I took on more child care despite also working full time.” Another wrote: “My husband works outside of the home and I have moved to 100% remote. I am now responsible for daycare and school drop offs and often pick ups- even when they interfere with my work day.” Mothers also reported a lack of support around child care and household chores from their partner, and several fathers also indicated that they weren’t supporting their partner. For example, one father wrote: “My wife is great. She does tell me I’m pretty lazy sometimes and has to ask me to help.”

The second subtheme related to changes in division of labour is *more shared home responsibilities between parents.* Parents reported that because both parents were home together more frequently, this allowed for child care and household responsibilities to become more collaborative. One mother wrote “He's been laid off since March 2020 so has been way more involved and takes care of the children while I work”. Several mothers who were stay-at-home parents before the COVID-19 pandemic also indicated that since their partner was home more, they were able to recognize and appreciate the effort required to manage child care and household tasks. One mother said: “Partner has been home more often so has been able to see the physical and emotional toll parenting can take.”

The third subtheme related to division of labour is *challenges with routine and renegotiating division of labour.* In contrast to parents’ responses about being able to share more responsibilities, other parents expressed frustration about their partner being home more since this resulted in disruptions to routines among some families. One mother wrote: “During covid our routine changed and my husband was home a lot more. We’ve definitely had hiccups with it in terms of housework division/priority, and how much he’s around in general.” Mothers also described the challenges of having to renegotiate logistics around child care and parenting styles with their coparent.

### Theme 2: More Hostility Between Parents

The second theme we found was increased hostility stemming from various challenges related to the COVID-19 pandemic which resulted in disagreements or arguments among parents. Some parents described hostility due to being “couped” up in the house with their partner and children. One father wrote: “We’re constantly in each other space for work and/or play and/or relaxation.” Parents, mostly mothers, also indicated that inequitable changes to division of labour also resulted in hostility between coparents. One mother wrote: “It hasn’t really changed except for when my partner was laid off for 3 months. He would spend time playing video games instead of helping me with the house even though he was the one who was home all day and night.”

### Theme 3: Increased Teamwork Among Parents

The third theme captured parents’ expressions about how the COVID-19 pandemic led parents to work together more effectively and develop stronger communication skills. One parent wrote: “We had to constantly communicate our schedules in order to balance various priorities. I think our relationship became stronger since we were communicating more.” Parents expressed how they had to be more coordinated to manage work, child care, and household responsibilities: “Without having access to activities and programs has forced us to become better at planning together.” Several parents mentioned having to “tag-team” or “trade-off” parenting in order to balance both child care and work. They discussed their strategy of having one parent manage child care while the other works and then switching to allow the other parent to work. Many parents highlighted how the challenges brought on by the COVID-19 pandemic necessitated increased teamwork among coparents, however several parents also mentioned that this benefit to their coparenting relationship was not without consequences to other aspects of their relationship and family: “Less cohesive family time. More trading off and on with child care so the other parent can complete work or house tasks.”

### Theme 4: More Family Time, but Less Time for Couples’ Relationship

The fourth theme reflected parents’ dichotomous comments about how the COVID-19 pandemic created more family time together but limited the quality time couples were able to spend alone together. Several parents expressed gratitude for the extra family time together and viewed this time as a silver lining of the pandemic: “We have spent more quality time with our family in the last year than the previous three years, but it is a good thing we work well together!” However, parents also mentioned that the increase in family time, time spent on child care and homeschooling, and changes to work hours meant that parents did not have as much time to spend together alone, without their children: “we had virtually no alone time together as one of us was always working which became very stressful and depressing.”

### Theme 5: More Stress Among Parents

The final theme captured parents’ comments about elevated levels of stress resulting from various COVID-19-related consequences. Parents of high-risk children mentioned the stress of navigating decisions about what’s best for their family while ensuring the safety and health of their child: “My partner is more stressed because she is always home with our child and can’t go anywhere do to our child being high risk.” Parents also commented about stress related to the unpredictability of child care because of daycare closures and illness: “stress related to who cares for child in case of daycare closures” Financial stress was also a key concern since many families experienced job loss or one parent having to quit their job to care for children: “More stress especially after spouse lost his job because of the pandemic.”

## Discussion

This embedded mixed methods study aimed to examine parents’ coparenting quality during the COVID-19 pandemic by comparing mothers’ and fathers’ CRS scores, and to explore mothers’ and fathers’ perceptions of changes in coparenting quality due to the COVID-19 pandemic by conducting a thematic analysis of their responses to an open-ended survey question. Overall, the main findings of the quantitative and qualitative analyses were in alignment and highlight how the unpredictable changes to work, child care, and home life due to COVID-19 had a differential impact on mothers’ and fathers’ coparenting quality. The quantitative results identified that, compared to mothers, fathers reported a more equal division of parenting duties and a more positive perception of their partner’s parenting, whereas mothers reported more undermining from their partners as compared to fathers. The thematic analysis provides useful context to these quantitative results identifying both similarities and differences in how mothers and fathers perceive how COVID-19 influenced their roles in the family and their relationship. While mothers and fathers both reported improvements to their communication and teamwork as well as a distancing in their relationship, mother and fathers differed in their reports of how they perceived their partners’ parenting and division of labour.

Overall fathers perceived a more equal division of parenting responsibilities compared to mothers, as demonstrated by their higher scores on the division of labour CRS subscale and their comments on the open-ended coparenting question about being more available to help with household tasks and child care. This finding aligns with prior quantitative and qualitative research highlighting division of labour as a key component of coparenting that was adversely affected by the COVID-19 pandemic (Carlson et al., 2022; Cox et al., 2023; Petts et al., 2021; Shafer et al., 2020). While some parents expressed that the COVID-19 pandemic resulted in more shared labour between parents, many other parents, and specifically mothers, reported that they felt disproportionately responsible for managing child care challenges due to daycare and school closures. Research from early in the pandemic (May 2020) found that fathers were more likely than mothers to report a more equal division of labour (Shafer et al., 2020). Fathers may have perceived a more equal division of labour because in the early months of the COVID-19 pandemic families were home more and fathers had more opportunity to engage in more child care and home tasks (Shafer et al., 2020). However, reports from parents in the United States have demonstrated that division of housework between mothers and fathers reverted back to pre-pandemic levels by fall 2020 (Carlson et al., 2022).

While fathers may have perceived a more equal division of parenting responsibilities as compared to mothers, responses from mothers reflected some frustration with how child care responsibilities fell largely on their shoulders. Prior research in Canada and in other countries has demonstrated similar findings, with mothers often bearing the responsibility of handling child care during COVID-19, despite also working the same number of hours as their partner (Calarco et al., 2021; Del Boca et al., 2021; Sevilla & Smith, 2020; Shafer et al., 2020). Reports of disproportionate and inequitable division of labour, along with elevated levels of stress reported from parents have significant implications for mothers’ mental health and careers. During the COVID-19 pandemic, a gap in psychological distress between mothers of young children and women without young children emerged, likely due to the increase in child care responsibilities among mothers (Childress, LaBrenz, et al., 2023; Zamarro & Prados, 2021). Additionally, since mothers remained the default parent in most different-sex couples, mothers’ careers were impacted as they had to take time off from work or pass on potential career opportunities in order to manage child care responsibilities (Petts et al., 2021). Mothers in the current study identified how they had to reorganize their work schedules since they felt responsible for managing child care and/or their partner did not have flexibility with their work schedule.

This study also explored other aspects of coparenting quality beyond division of labour, which have received limited attention in the context of the COVID-19 pandemic. Fathers scored higher than mothers on endorsement of partner’s parenting, indicating that fathers had a more positive perception of their partner’s parenting as compared to mothers. This result was also reflected in the thematic analysis where several fathers commented on how being home more allowed them to gain a better appreciation for their partners’ parenting and home responsibilities. Prior research, conducted before the COVID-19 pandemic, has found similar findings, with fathers perceiving their partners more positively than mothers, and with mothers being more critical of fathers’ parenting (Allen & Hawkins, 1999; Gallegos et al., 2019). Mothers also reported more undermining from fathers on both the CRS and in the thematic analysis, possibly due to fathers being home more and being more involved in child care tasks. Fathers’ increased involvement required parents to renegotiate their parenting styles and communication in the context of their new routines. To support fathers’ involvement, parenting information and supports should prioritize targeting and including fathers in their messaging. Additionally, helping parents overcome their perceptions of gender-stereotypes in parenting may help guide them toward a more child-centered coparenting approach, rather than on a parent-centered individualistic approach (Gallegos et al., 2019).

We found no significant differences between mothers and fathers on the agreement, closeness, support, and conflict subscales of the CRS. Findings from the thematic analysis show that both mothers and fathers reported feeling less emotionally close with their partner because of the lack of couples-only time, more hostility in their relationship stemming from pandemic-related challenges, and the need for more teamwork and coordination between parents. These results are consistent with findings from a qualitative study by Cox et al. (2023) who found that mothers reported that during COVID-19 they received less support from their partner, but also experienced improved communication and collaboration with their partner. Lucassen et al. (2021) measured similar aspects of coparenting quality and found that higher levels of stress pre-pandemic and greater increases in stress from pre-pandemic to during the lockdown in the Netherlands predicted lower levels of coparenting quality during the lockdown, and these associations did not differ between mothers and fathers. Certain aspects of coparenting have been shown to moderate or mediate associations between pandemic-related stressors and family and parenting outcomes. For example, greater support and cooperation between coparents can buffer the effect of parental distress on harsher parenting (McRae et al., 2021), whereas health stress has been associated with greater conflict between coparents which is subsequently associated with a decrease in family functioning (Peltz et al., 2021). Therefore, understanding how individual components of coparenting quality are affected by large-scale disruptions is important as these aspects of coparenting could be included in future parenting interventions or supports to help families mitigate the negative effects of disruptions on their families and relationships. More longitudinal research is needed to understand shifts in coparenting quality among mothers and fathers from pre-pandemic through the next several years and how various program or policy supports may impact partners’ coparenting.

Findings from this study highlight mothers as being particularly at risk for lower coparenting quality due to shifts in childcare and household responsibilities during the COVID-19 pandemic. Future policy level initiatives aimed at minimizing the impact of the COVID-19 pandemic should prioritize supports for mothers’ mental health and aim to address the inequitable distribution of childcare and household responsibilities between mothers and fathers. For example, Javed et al. (2019) found that policies that allowed for reduced work hours, working from home, and limiting work to school hours were associated with less anxiety and depression among married mothers. Given that mothers are more likely than fathers to take on the childcare responsibilities, policies to support access to innovative and affordable childcare can help support mothers in maintaining balance between their work and family responsibilities (Bariola & Collins, 2021; Childress, Roberts, et al., 2023). Organizations should also prioritize efforts to diversify leadership, including by recruiting women into leadership roles. By having women representing in positions of leadership, companies can work towards developing policies that represent the needs of all individuals within their organization (Profeta, 2021). Finally, encouraging fathers to take time off for childcare or to work from home could potentially decrease the pressure on mothers to act as the default parent among different-sex couples.

To our knowledge, this is the only study that used an embedded mixed methods design to examine coparenting quality during COVID-19 among both mothers and fathers. Using this mixed methods approach allowed for richer insights into coparenting that could be reached using only quantitative or qualitative methods (Johnson & Onwuegbuzie, 2004) and including both fathers and mothers allowed for both parents’ perspectives to be explored providing a more comprehensive understanding of family-level factors that have important influences on both child and parent outcomes (Broderick, 1993; Feinberg, 2003). There are also limitations that should be considered when interpreting the results of this study. First, parents completed surveys between July 2020 to December 2021, therefore the long-term impact of the COVID-19 pandemic on coparenting quality requires further investigation. Second, some parents commented that since they became parents shortly before or at the beginning of the pandemic, it was difficult to distinguish between coparenting challenges due to COVID-19 and challenges of early parenthood. Additionally, most parents identified as white, were in different-sex relationships, and graduated college or university, therefore findings might not be generalizable to other races or ethnic groups, same-sex coparenting relationships, or parents with different levels of education. Finally, while our thematic analysis of open-ended survey items provides a more in-depth understanding of parents’ perceptions of coparenting than can be obtained from close-ended survey items, this data collection approach does not allow for probing or follow-up questions from researchers which limits the level of detail in participants’ responses.

## Conclusion

In this study of parents’ coparenting quality during the COVID-19 pandemic, we found that, compared to mothers, fathers perceived a more equal division of labour and had a more positive perception of their partner’s parenting, and mothers reported more undermining from their partners compared to fathers. The thematic analysis highlighted five key themes: gendered changes to division of labour, more hostility, more teamwork, less alone time for couples, and more stress among parents, particularly among mothers. Results of this study reinforce previous research identifying mothers at being particularly at risk for mental health challenges and career lapses due to shifts in child care and household responsibilities during the COVID-19 pandemic (Bastiaansen et al., 2021; Gordon & Presseau, 2023; He et al., 2021). As research and policy efforts continue to develop to support families in recovering from the pandemic, addressing more equitable division of labour and overall coparenting quality should be considered as a potential mechanism for mitigating adverse outcomes on families.
